# Alternative therapy in glaucoma management: Is there any role?

**DOI:** 10.4103/0301-4738.73679

**Published:** 2011-01

**Authors:** Rajul S Parikh, Shefali R Parikh

**Affiliations:** 1 Shreeji Eye Clinic & Palak’s Glaucoma Care Centre, Mumbai, India; 2 Bombay City Eye Institute & Research Centre, Mumbai, India; 3 Dr. L H Hiranandani Hospital, Mumbai, India; 4 Lotus Eye Hospital, Mumbai, India

**Keywords:** Alternative and complementary therapy, dietary antioxidants, glaucoma, intraocular pressure, life style

## Abstract

Glaucoma is one of the leading causes of blindness worldwide. Various randomized controlled clinical trials have shown that lowering intraocular pressure (IOP) does reduce progression of primary open-angle glaucoma. However, there is lots of interest in nonpharmacological options that includes lifestyle adjustment and alternative and complementary therapy (ACT). At least 5% glaucoma population uses ACT. Various lifestyle activities like exercise and alcohol can reduce IOP by 1 to 2 mm Hg but would have small effect on glaucoma. The psychological stress can increase IOP. Hypothetically and few studies do show neuroprotective effect (or effect on ocular blood flow) of alcohol, *Gingko biloba*, bilberry, but the current evidence is weak for its routine use. We must also remember the side effects of ‘medications’ (e.g., marijuana, alcohol) before promoting as remedy for glaucoma. In current armamentarium of glaucoma management, ACT cannot substitute the conventional treatment available to lower IOP.

Doctor, is there anything else that I can do, other than putting medicines regularly, to prevent my eyes from becoming blind due to glaucoma?

No doubt, every ophthalmologist must have faced this intricate question again and again. Patients often asks about availability of alternative and complementary therapy (ACT) for glaucoma (due to improvement in patient awareness and easy access to internet). Definitely, we do not have enough scientific data to answer them affirmatively, but if we ignore their question as scarce literature available on internet may mislead them.[1] ACT for glaucoma refers to the disease management strategies other than conventional therapy, that is, pharmaceutical, laser, or surgical treatment known to lower intraocular pressure (IOP). A survey in an urban area of United Kingdom reported use of ACT by 5% of the patients; 60% of these would use more than one option for glaucoma management.[1]

Weak evidence suggests that certain lifestyle activities may elevate IOP and thus could predispose to primary open-angle glaucoma (POAG) in subset of patients. Detailed history of personal habits and lifestyle has helped to solve some complicated progressive glaucoma patients with otherwise normal IOP on diurnal variation tests. A person playing high wind musical instruments (eg, Saxophone) can have two-fold increase in his IOP from baseline level for short duration.[2] Though there is no strong evidence that playing these instruments predisposes to glaucoma, it is reasonable to inform a glaucoma patient who play these instruments about this effect. Certain yoga exercises in inverted position (like Shirshasana) leads to two- to three-fold IOP rise from baseline for short duration.[3] We documented a three-fold increase in IOP during the inverted position (IOP checked with Tonopen?) in at least three of our progressive POAG patients with good 24-hour IOP control (personal communication with Dr. Rajul S Parikh). Though evidence is not sufficient to prove the role of inverted yoga exercises as a causal risk factor for glaucoma progression, it is imperative to inform glaucoma patients about IOP elevations associated with inverted posture.

A prolonged stress-induced increase in endogenous cortisol and catecholamines with subsequent alterations of the immune response may increase IOP.[4,5] So, it may be prudent to ask about potential psychosocial or environmental stress factors, especially in a patient who had stable disease and now has developed a dramatic rise in IOP or deterioration of visual function.

Caffeine is consumed by a high percentage of the general public. Most of the studies,[6,7] with some exceptions,[8] demonstrate 2 to 3 mm Hg increase in IOP that lasts for about 2 hours after use of coffee. However, coffee beans also contain compounds that have antioxidative effects.[9,10] These antioxidative effects and its possible neuroprotective implications need further research.

Literature also suggests the variable effect of alcohol on IOP and its role in neuroprotection. Weak evidence indicates that alcohol consumption leads to dose-related IOP reduction which may last for several hours through temporary osmotic effect.[11–13] Intake of large quantities of beer or water definitely increases IOP significantly due to volume overload (mimicking water drinking test). However, a few other studies did not report any association between alcohol intake and IOP.[14,15] ‘Neuro-protective’ effect of red wine has also been reported. Though consuming one alcoholic drink per day may have some cardiovascular benefits,[16] it also increases the risk of liver disease.[17] As evidence is not sufficient enough to prove the beneficial effects of alcohol on glaucoma, it is important to discourage the belief that drinking alcohol will reduce the risk of glaucoma. Moreover, we do not want people to initiate drinking at the age of 60 years to prevent ‘blindness’ and get cirrhosis. *‘Have Wine, keep your nerve healthy and prepare yourself for liver transplant.’*

Similarly, literature showing the variable effect of exercise on IOP is available. Few studies reported that aerobic exercise causes lowering of IOP,[11,18–20] while few other studies concluded that isometric exercise like lifting weights may produce a small IOP increase during exertion.[21] Certain subtypes of glaucoma like pigmentary glaucoma may have IOP rise after exercise. However, it remains uncertain whether such exercise-induced IOP changes correlates with glaucoma pathogenesis and/or progression. Currently, there are no cohort studies reporting the relationship between exercise and glaucoma; moderate aerobic exercise has many health benefits and thus should be encouraged.

Recently, there is a considerable interest in dietary antioxidants (e.g., red wine, dark chocolate, coca, green tea, curcumin, glutathione, n-6 to n-3 polyunsaturated fatty acid, etc) because oxidative stress may induce damage to the outflow channel as well as to the optic nerve.[22] However, there is no strong evidence suggestive of beneficial effect of dietary antioxidant intake and its neuroprotective effect on POAG;[23] further research is needed in this direction. At this time, one cannot promote antioxidant intake as a strategy to prevent the development of POAG or slow down its progression. Curcumin (used widely in India) having antioxidative property has shown possible beneficial effects on altering various mechanisms like oxidative stress, excitotoxicity, aberrant immunoregulation, neurotrophin deprivation, or abnormal TNF-α signaling.[24]

Marijuana, a cannabinoid, has been proposed as IOP-lowering agent for a long time. The active ingredient in marijuana (delta-9-tetrahydrocannabinoid) reduces IOP by reducing aqueous humor production.[25,26] IOP-lowering effect of marijuana is for short term and one needs to smoke marijuana every 3 hours for 24-hour IOP control.[27] *‘Keep smoking, start hallucinating and keep your IOP under control.’* Fortunately, use of marijuana even for medicinal purposes is illegal in India. Overall, the existing evidence suggests that use of marijuana for glaucoma management is not a feasible option either for medical or legal reasons, as it may actually do more harm to other parts of the body. Recently, there is surge in interest in newer topical synthetic cannabinoid receptor agonist, WIN 55211-2 as it lowers IOP in rats, monkeys, and human beings with a tolerable side-effect profile.[28]

*Gingko biloba* and bilberry (shrubs that yield a fruit resembling blueberries) have been proposed as neuroprotectors. The main components of the *Gingko* leaf extract are flavonoid glycosides and terpene lactones. Gingko is thought to mediate its effects through several biological mechanisms including antiplatelet action, vasodilation, and antioxidant effect. There is little data regarding the effect of *Gingko biloba* on the course of glaucoma. A placebo-controlled randomized controlled trial (RCT) found that *Gingko biloba* improved preexisting visual field loss in some patients with normal tension glaucoma.[29,30] Further research is mandatory before we start recommending use of ginkgo biloba along with conventional glaucoma therapy on a routine basis. Bilberry extracts contain high quantities of anthocyanin, a flavonoid with antioxidant properties. Various other herbs and plants have been proposed in various parts of the world and they are discussed in recent World Glaucoma Association Consensus book on medical management of open angle glaucoma.[30]

Acupuncture is also being used in different parts of the world as one of the alternative therapy for management of glaucoma. A Cochrane review in 2007 concluded that there is no useful data regarding the effect of acupuncture on glaucoma.[31] Patients should be counseled regarding the paucity of high-quality evidence on the beneficial effect of acupuncture in glaucoma and other diseases.

To conclude, when patients inquire about the relationship between lifestyle factors, alternative medicine, and glaucoma, the physician should take the opportunity to educate them about their disease. Few activities should be avoided by the glaucoma patients, although more evidence is needed to determine if these activities predispose to glaucoma or contribute to the progression of the pre-existing disease. There are no RCTs that assess whether ACT may prevent glaucoma or slow down the disease progression. When counseling patients about ACT, it is important to know which forms of therapy are not feasible or might be harmful. It is also important to emphasize that in current armamentarium of glaucoma management, ACT cannot substitute the conventional treatment available to lower IOP. We need to answer lots of questions before disregarding ‘alternative medicine’ or start using them along with our ‘standard care.’
